# Drugs and Nutrients in Epilepsy: Vitamin B6 and the Ketogenic Diet

**DOI:** 10.3390/nu17162676

**Published:** 2025-08-19

**Authors:** Shani Bahalul-Yarchi, Feigy Hartman, Karin Ben Zaken, Ibrahim O. Sawaid, Lior Segev, Samuel Mesfin, Pnina Frankel, Rahaf Ezzy, Abraham O. Samson

**Affiliations:** Azrieli Faculty of Medicine, Bar Ilan University, Safed 1311502, Israel

**Keywords:** nutrients, epilepsy, vitamins, vitamin B6, ketogenic diet

## Abstract

Certain foods and specific drugs have been linked to epilepsy in the literature. Here, we query PubMed citations for the co-occurrence of epilepsy with foods and drugs, using a list of 217,776 molecules from the HMDB. Notably, the top associations with epilepsy include approved drugs and drug families, diagnostic markers, inducers, and vitamins. Drugs include fosphenytoin (40%), topiramate (37%), valproic acid (34%), hydantoin (20%), phenytoin (31%), carbamazepine (33%), carbamazepine-10,11-epoxide (40%), trimethadione (31%), gabapentin (14%), pregabalin (11%), flunarizine (7%), fenfluramine (4%), bumetanide (4%), KBr (18%), cannabidiol (14%), clonazepam (22%), nitrazepam (10%), diazepam (7%), lorazepam (6%), midazolam (3%), amobarbital (21%), phenobarbital (16%), flumazenil (7%) allopregnanolone (7%), pregnanolone (6%), epipregnanolone (6%), 3-hydroxypregnan-20-one (6%), and vitamin B6 (6%). Drug families and scaffolds include imidazolidine (18%), succinimide (10%), acetamide (7%), 2-pyrrolidinone (7%), pyrrolidine (6%), tetrahydropyridine (6%), and isoxazole (4%). Investigational compounds include cyano-7-nitroquinoxaline-2,3-dione (5%). Diagnostic markers include exametazime (10%) and quinolinic acid (3%). Inducers include flurothyl (37%), pentetrazol (32%), pilocarpine (25%), (+)-Bicuculline (8%), and 1-methyl-4-phenyl-1,2,5,6-tetrahydropyridine (MPTP, 6%). Our analysis highlights frequently cited associations between epilepsy and specific drugs and highlights the importance of supplementing nutrients with vitamin B6 and the ketogenic diet, which increases the gamma-aminobutyric acid (GABA)/glutamate ratio. As such, our study offers dietary approaches in the treatment of this neurodegenerative disease.

## 1. Introduction

Epilepsy is commonly associated with several neurodegenerative and pathological alterations in areas of the brain that are involved in repeated electrographic seizures [1]. While the exact cause of epilepsy is sometimes unknown, it can arise from several factors like central nervous system (CNS) injury, stroke, tumors, infections, or birth defects [2]. The manifestation of both clonic and tonic seizures is linked to distinct anatomical features and heightened neuronal activity. Genetic mutations have been linked to a small number of cases and involve multiple genes [3]. Current therapies for epilepsy aim to control seizures and to improve the quality of life for patients. Primary treatment includes antiepileptic drugs (AEDs) like valproate, carbamazepine, and newer agents such as levetiracetam and lamotrigine [4]. For those with drug-resistant epilepsy, surgical options like lobectomy, lesionectomy, or vagus nerve stimulation (VNS) are elective [5]. Recently, responsive neurostimulation and deep brain stimulation have shown promise in some refractory cases [6]. Advances in genetics and personalized medicine are also paving the way for more tailored and effective treatments [7].

Nutrients play a key role in epilepsy, and certain foods, or a lack thereof, increase the risk of epileptic seizures. Dietary approaches, such as the ketogenic diet, involving a high-fat, low-carbohydrate approach, have proven beneficial, particularly for children whose seizures remain unresponsive to anti-seizure medications [8]. About half of children on a ketogenic diet experience a 50% reduction in seizures, and about half achieve a reduction of more than 90% [9]. Caffeine intake may also be a factor to consider when achieving and maintaining seizure control in epilepsy. Coffee has increased seizure susceptibility in animal models, but it has also shown protective effects against seizures. At present, clinical data in humans and the impact of coffee on epileptics is insufficient [10]. Alcohol, when taken responsibly, is safe for most patients with epilepsy [11]. Vitamin D deficiency has been shown to play a role in the pathophysiology of epilepsy [12]. Notably, vitamin B6 (i.e., pyridoxine) provides rapid control in patients with various conditions affecting pyridoxal phosphate (PLP) synthesis and recycling [13]. Additionally, vitamin B6 is effective in conditions where metabolites accumulate and inactivate PLP, such as Aldehyde Dehydrogenase 7 Family Member A1 (ALDH7A1) deficiency and hyperprolinemia type II. Magnesium deficiency can occasionally cause epilepsy, and recognizing and correcting this deficiency may be life-saving [14]. Gluten sensitivity or celiac disease has been linked to increased seizure activity in susceptible individuals, and these patients may benefit from a gluten-free diet [15]. Omega-3 fatty acid deficiency has also been linked with the risk of epilepsy, and these patients benefit from including omega-3 supplements in their diet [16]. The role of zinc in seizures is controversial because, on one hand, it aids in the synthesis and function of the inhibitory neurotransmitter γ-aminobutyric acid (GABA), but on the other hand, it also inhibits GABA, which can facilitate seizure activity [17]. Vitamin B12 deficiency, and high folate levels, have been shown to cause generalized tonic–clonic seizures in rare cases, highlighting an unusual neurological condition linked to these vitamin imbalances [18]. Finally, electrolyte imbalances, such as hypocalcemia, hyponatremia, and hypomagnesemia, can also manifest with acute symptomatic seizures [19].

Bioinformatics uses a multitude of information to analyze medical data. Bioinformatic techniques, such as text mining and citation counts, are often used to identify trends and patterns in medicine [20]. These are powerful technologies for quickly distilling key information from vast quantities of biomedical literature [21]. Several studies have used text mining, and notably, Bork et al. captured the phenotypic effects of a drug based on the side effects resources published by the Food and Drug Administration (FDA) [22]. In another study, Jensen and coworkers used text mining to associate diseases and genes and to establish a web-based database named DISEASE [23]. In the past, we have used frequency analysis of PubMed citations and have shown that antibiotic resistance is cyclic [24,25]. Furthermore, we have used PubMed frequency analysis to classify autoimmune diseases [26]. Finally, we have used PubMed frequency analysis to find comorbid conditions with Alzheimer’s disease [27]. Currently, some 50 million people worldwide are affected by epilepsy, and more than 225,000 PubMed citations are related to epilepsy, thus providing a wealth of information [28].

In this review, we use citation mining to systematically rank and highlight key compounds associated with epilepsy in the literature. Then, we classify the compounds associated with epilepsy and discuss their potential benefit, or detriment, in the context of epilepsy.

## 2. Methods

### 2.1. List of Nutrients

To prepare a list of nutrients, we downloaded a comprehensive list of 217,776 molecules from the Human Metabolome Database (HMDB 5.0) [29].

### 2.2. PubMed Count and Epilepsy Association

To mine for molecule associations with epilepsy, we queried PubMed for molecule terms using a Python (3.12) script. First, the program counted the number of PubMed co-citations with epilepsy and the molecule term (e.g., “epilepsy” AND “resveratrol”) and for the molecule alone (e.g., “resveratrol”). Then, to normalize the association, the program divided the number of co-citations of the molecule alone. The normalization considered the relative abundance of popular molecules over the relative dearth of rare molecules. Finally, the normalized association was multiplied by 100 to obtain a percentage value. The normalized PubMed association corresponded to the following generalized formula:

$${Normalized\;Association}_{Molecule+Epilepsy}\;=\;\frac{\left[C{itations}_{Molecule+Epilepsy}\right]}{\left[{Citations}_{Molecule}\right]}\;\times \;100$$

To obtain accurate results for multi-word terms, parentheses with double quotes were used in all our searches. Our algorithm performed ~1600 searches per hour and printed the normalized associations in a text file that could be imported into Excel for easy sorting.

## 3. Results

**Figure 1:**
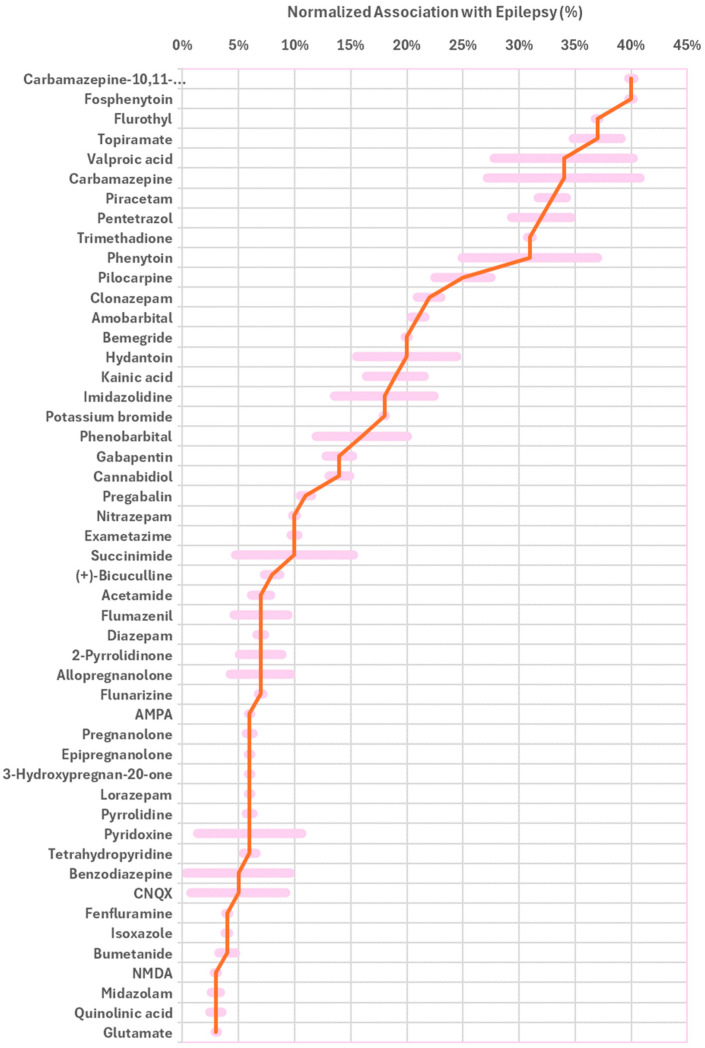
**Foods and drugs associated with epilepsy.** Shown are the top normalized associations (percent value, red line) and co-citations (arbitrary units, pink bars) with epilepsy according to PubMed. Associations are not indicative of causation, and do not reflect whether correlation is positive or negative.

Here, we query PubMed citations for the co-occurrence of epilepsy with foods and drugs, using a list of 217,776 molecules from the Human Metabolome Database (HMDB 5.0) [29]. Figure 1 shows the top molecules associated with epilepsy, above an arbitrary threshold of 3%, and more than 100 co-citations. Notably, the top classifications include drugs used in the treatment of epilepsy, diagnostic markers, and inducers of epilepsy in animal models. The top associations are classified according to function, and their nutritional benefits are discussed in the following section.

### 3.1. Antiepileptic Drugs (AEDs)

Trivially, the top associations include antiepileptic drugs (AEDs), both chronic and acute. Chronic AEDs are used for the long-term control and prevention of seizures, while acute AEDs are used to quickly stop or reduce the severity of status epilepticus. Chronic AEDs include sodium channel blockers, calcium channel blockers, and halides, as well as GABA, AMPA, and cannabis derivatives. Sodium channel blockers include fosphenytoin (40%) [30], topiramate (37%) [31], valproic acid (34%) [32], hydantoin (20%), phenytoin (31%) [33], carbamazepine (33%), and its active metabolite carbamazepine-10,11- epoxide (40%) [34]. Notably, the active metabolite carbamazepine-10,11-epoxide (40%), which is used for the therapeutic monitoring of carbamazepine treatment, can also lead to clinical toxicity [35]. Calcium channel blockers include trimethadione (31%) [36], gabapentin (14%) [37], pregabalin (11%) [37], and flunarizine (7%) [38]. Flunarizine has a weak effect on seizure frequency and has a significant withdrawal rate, probably due to side effects, and is not recommended for use as an adjuvant therapy. Derivatives of the neurotransmitter γ-aminobutyric acid (GABA) include piracetam (33%) [39]. Halides include potassium bromide (KBr, 18%). KBr is a halide anticonvulsant that is particularly effective for tonic seizures, generalized tonic–clonic seizures, and secondary generalized seizures, linked with pediatric refractory epilepsy [40]. Cannabis derivatives include cannabidiol (14%), a modulator of the cannabinoid receptors. Cannabidiol has been shown to reduce monthly seizure frequency by 36.5% in children and young adults with highly treatment-resistant epilepsy, but not without adverse effects [41]. Fenfluramine (4%) is a serotonergic agent and is effective in the treatment of epilepsy in Dravet syndrome (DS) and Lennox–Gastaut syndrome (LGS) [42]. Bumetanide (4%) is an FDA-approved potent loop diuretic used off-label in the treatment of temporal lobe epilepsy [43]. Bumetanide is an antagonist of sodium-potassium-chloride cotransporters, expressed in the CNS. Notably, many of the aforementioned AEDs have multiple modes of action, such as gamma-aminobutyric acid (6%) receptor agonism, glutamate (3%) receptor antagonism, N-methyl-D-aspartic acid (NMDA, 3%) receptor agonism, and alpha-amino-3-hydroxy-5-methyl-4-isoxazolepropionic acid (AMPA, 7%) receptor antagonism. Likewise, GABA, NMDA, glutamate, and AMPA derivatives also play an important role in the treatment of epilepsy.

The top associations also include AEDs such as benzodiazepines (5%) and barbiturates mainly used for the acute treatment of epilepsy, as well as the benzodiazepine antidote, flumazenil. Benzodiazepines include clonazepam (22%), nitrazepam (10%), diazepam (7%), lorazepam (6%), and midazolam (3%), and barbiturates include amobarbital (21%) and phenobarbital (16%) [44]. Benzodiazepines are often preferred over barbiturates in the treatment of status epilepticus. Despite the relatively good safety profile of phenobarbital, barbiturates have no antidote [45]. Notably, intranasal transmucosal delivery of benzodiazepines is useful in reducing time to drug administration and the cessation of seizures in the pre-hospital setting, when actively seizing patients arrive in the emergency room, and at home where caregivers treat their dependents [46]. Flumazenil (7%) is not typically used in the treatment of epilepsy. Instead, it is primarily used as an antidote to reverse the effects of benzodiazepine overdose or to wake patients following benzodiazepine sedation. Paradoxically, flumazenil has been shown to possess anticonvulsant properties [47], yet it can also cause convulsions [48]. While flumazenil does not reduce the antiepileptic effect of diazepam [49], it can induce seizures after benzodiazepine administration [50]. These inconsistencies might be due to variations in dosage and the type of seizure involved and highlight the need for further clinical investigation. In the meantime, flumazenil should be used with extreme caution in epileptic patients.

The top associations also include allopregnanolone (7%), pregnanolone (6%), epipregnanolone (6%), and 3-hydroxypregnan-20-one (6%). Allopregnanolone is an endogenous steroid and a positive modulator of GABA_A_ receptors with antiseizure activity. Allopregnanolone administered intranasally has been shown to confer rapid seizure protection and could have potential in the treatment of seizure emergencies [51].

Finally, the top associations also include pyridoxine (6%), better known as vitamin B6. Pyridoxine provides rapid control in patients with *pyridoxine-dependent epilepsy*, which includes various conditions affecting pyridoxal phosphate (PLP) synthesis and recycling [11]. Pyridoxine-dependent epilepsy is a rare autosomal recessive disorder, classically presenting with neonatal seizures that can be controlled with pharmacologic doses of pyridoxine [52].

### 3.2. Antiepileptic Drug Families and Scaffolds

The top associations include anticonvulsant family names and molecular drug scaffolds used in epilepsy, such as imidazolidine (18%), succinimide (10%), acetamide (7%) 2-pyrrolidinone (7%), pyrrolidine (6%), and tetrahydropyridine (6%). Imidazolidine (18%) is a five-membered ring composed of three carbons and two non-adjacent nitrogens with the chemical formula C_3_H_8_N_2_. Imidazolidine is the pharmacological scaffold of fosphenytoin, phenytoin, hydantoin, amobarbital, and phenobarbital, and it has been used to develop potential AEDs [53]. Succinimide (10%) is a five-membered ring comprising one nitrogen flanked by two carbonyls, with the chemical formula C_4_H_5_NO_2_. Succinimide is the pharmacological scaffold of fosphenytoin, hydantoin, and phenytoin, and it is also the family name of *succinimide* anticonvulsants, such as phensuximide and methosuximide. While the latter accounts for most of its co-citations with epilepsy, the succinimide ring has also been used to develop potential AEDs [54]. Acetamide (7%) anticonvulsants include piracetam (33%) and levetiracetam. In addition, acetamide (7%), 2-pyrrolidinone (7%), pyrrolidine (6%), tetrahydropyridine (6%), and isoxazole (4%) are other pharmacological scaffolds that have been used in the development of potential AEDs [55,56,57].

### 3.3. Diagnostic Markers

The top associations include markers compounds used in neuroimaging, such as exametazime (10%). Exametazime (10%), also known as hexamethylpropyleneamine oxime, complexed with radiolabeled ^99m^Tc is used as part of single-photon emission computed tomography and positron emission tomography techniques to determine the seizure ictal onset zone, which needs to be resected to render a patient seizure-free [58]. In addition, quinolinic acid (3%) is also a biomarker of neuroinflammation in epilepsy [59].

### 3.4. Biomarkers for GABA-Transaminase Deficiency

The top associations include biomarkers for GABA-transaminase deficiency, such as succinimide (10%) and 2-pyrrolidinone (7%). This neurological disorder leads to GABA accumulation in the cerebrospinal fluid (CSF), causing severe developmental delay, intellectual disability, seizures, and movement disorders, often resulting in early childhood death. Diagnosis primarily involves measuring GABA in CSF, but elevated levels of 2-pyrrolidinone, succinimide (or its ring-opened form, succinamic acid), and homocarnosine are also used for clinical screening [60].

### 3.5. Inducers of Epilepsy in Animal Models

The top associations also include drugs used to induce epilepsy in animal models, such as flurothyl (37%), pentetrazol (32%), (+)-bicuculline (8%), pilocarpine (25%), and 1-methyl-4-phenyl-1,2,5,6-tetrahydropyridine (MPTP, 6%). Flurothyl (37%) is an inhaled antagonist of the GABAergic system, which induces seizures within minutes [61]. Flurothyl-induced seizures end within 30 s of returning animals to room air, representing a useful model to study the effect of epileptic seizures. Another GABA receptor antagonist, pentetrazol (32%), administered intraperitoneally has also been used to induce kindling and epileptic seizures in animal models [62]. Bicuculline (8%), a phthalide-isoquinoline compound, is an antagonist of GABA receptors, and it has been used intraperitoneally to induce epilepsy in both adult and immature animal models [63]. Pilocarpine (25%) is a muscarinic agonist and pilocarpine-induced seizures consist of automatisms, Worster-Drought syndrome (WDS), clonic seizures, and status epilepticus (SE) [64]. 1-Methyl-4-phenyl-1,2,5,6-tetrahydropyridine (6%), better known as MPTP, is an acute proconvulsant, but has no long-term effects in animal models of seizures and epilepsy [65]. However, MPTP does present long-term effects in the same animal model of Parkinson’s disease upon injection into the substantia nigra [66]. The top associations also include kainic acid (19%). Kainic acid is an agonist of the glutamic kainate receptors, and systemic, intrahippocampal, and intranasal administration induces epileptic seizures in animal models [67]. The top associations also include bemegride (20%). Bemegride is a CNS stimulant that has been used as an activation agent in electroencephalography in animals [68]. It was popular in the 1950s and 1960s, coinciding with the peak of lobotomy, which is now discredited, but has since fallen into obscurity. Notoriously, bemegride was used in patients with suspected epilepsy, to help diagnosis [69]. Nowadays, bemegride use is mostly limited to veterinary medicine.

### 3.6. Investigational Compounds

The top associations also include investigational compounds, such as 6-cyano-7-nitroquinoxaline-2,3-dione (CNQX, 5%), which is an investigational new compound used in research to protect animal models from seizures, such as absence epilepsy [70]. CNQX is a competitive AMPA/kainate receptor antagonist.

## 4. Discussion

In this study, we explore the top molecules associated with epilepsy, using PubMed co-citations. The molecules are grouped according to function, and they include approved drugs, disease inducers, diagnostic markers, and vitamins. Approved antiepileptic drugs (AED) include fosphenytoin (40%) and topiramate (37%), among others. Neuroprotective compounds include vitamin B6, which protects against pyridoxine-dependent epilepsy. Epilepsy inducers in animal models include flurothyl (37%) and pilocarpine (25%). Diagnostic markers include exametazime (10%). Table 1 classifies the top molecules into indicated drugs, diagnostic markers, inducers, and others.

**Figure 2:**
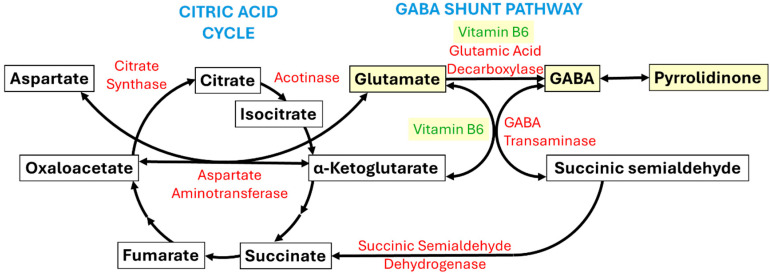
**GABA shunt pathway.** The gamma-aminobutyric acid (GABA) shunt pathway (right) connects to the citric acid cycle (left). Enzyme names are in red, substrate names are in black, and cofactor (i.e., vitamin B6) names are in green. Note that molecular intermediates that are dysregulated in epilepsy, namely glutamate, GABA, pyrrolidinone, and vitamin B6, are highlighted in yellow. In epilepsy, GABA, pyrrolidodinone, and vitamin B6 levels are low, while glutamate levels are high.

This study highlights the importance of the ketogenic diet and vitamin B6 (6%) supplementation in pyridoxine-dependent epilepsy. Recently, the ketogenic diet was shown to prevent epilepsy by producing β-hydroxybutyric acid, which augments brain levels of gamma-aminobutyric acid (GABA) and increases the GABA/glutamate ratio [71]. Figure 2 shows the GABA shunt pathway that intersects with the citric acid cycle. Notably, vitamin B6 plays an important role in GABA production, the main inhibitory neurotransmitter of the CNS. Furthermore, this study highlights the importance of avoiding seizure inducing medication, such as the antitussive drug pentetrazol, sold in Italy under the brand name Cardiazol-Paracodina, and containing a solution of succindihydrocodeine and pentetrazole. Likewise, preparations containing pilocarpine should also be avoided.

There is growing but incomplete evidence for sex-specific responses to both vitamin B6 and the ketogenic diet in epilepsy. For example, pentetrazole sensitivity is more pronounced in female rats than males, and prior administration of vitamin B1 and B6 delays seizures in female rats only [72]. Likewise, the ketogenic diet increases oxidative stress, cellular senescence [73], and glucose intolerance [74] in male, but not female mice. Interestingly, the increase in body weight, glucose levels, blood insulin levels, and histone modification associated with the ketogenic diet is more pronounced in male mice compared to female mice [75]. These differences appear to be driven by hormonal, metabolic, and neurochemical factors. However, more clinical studies are needed to tailor therapies based on gender. Importantly, in hospitals that treat epilepsy, including cancer centers, nutritionists are part of the care team. These nutritionists can readily identify dietary issues, such as gluten intolerance, and can recommend ways to correct nutrient deficiencies, including supplementation with vitamin B6 when needed [76].

### 4.1. Limitations

This study does not differentiate between the different types of epilepsy and seizure. Recently, the International League Against Epilepsy (ILAE) generated and updated the classification system for both seizures and epilepsies in 2017 [77]. The 2017 classification system includes formerly ignored seizure types and integrates the genetic underpinning of epilepsy etiology [78]. In addition, in 2022, the ILAE published consensus criteria, definitions, and categorization of the different epilepsy syndromes [79]. As our understanding of the genetic underpinnings of epilepsies increases, so does the prevalence of individualized precision treatments and personalized medical therapies. The genetic underpinnings of epilepsy have helped in the classification of these syndromes, and examples now include rare monogenic forms involving KCNA2 [80], KCNT1 [81], and SCN1A [82]. Despite these limitations, this study offers valuable insights and establishes a foundation for future research to further unravel the complexities of epilepsy and enhance targeted therapeutic strategies.

The normalized associations calculated herein are based on incidental co-citations in PubMed. As a potential limitation, the normalized association is ‘polluted’ by publications that randomly mention molecules and epilepsy. To offset this “pollution”, we only included molecules with at least 100 co-citations. Assuming random noise, we ignored any molecule with a signal-to-noise ratio below 10 (Signal = 100, Noise = √Signal = 10). Finally, to further reduce the chance of random co-citation, we queried only the title, abstract, and other terms of PubMed citations, not the full-text paper. These precautions significantly reduce random noise. As another potential limitation, normalized associations do not indicate causation, nor do they reflect whether the correlation is positive or negative. Also, normalized associations are time-dependent, and co-citation could refer to molecules that peaked decades ago and then faded into oblivion. To offset these limitations, great care was taken to only include timely and relevant studies and to only cite recent publications.

### 4.2. Conclusions

The potential dietary and pharmacological approaches discussed in this review require further validation through well-designed clinical studies. The present findings are based on literature trends and should not be interpreted as definitive clinical recommendations. Despite these limitations, this study highlights the importance of supplementing diets with vitamin B6, as well as the importance of the ketogenic diet, which increases the GABA/glutamate ratio in patients with epilepsy.

## Figures and Tables

**Table 1:** Classification of top associations.

Role in Epilepsy	Molecule
**Drug**	Fosphenytoin (40%), topiramate (37%), valproic acid (34%), hydantoin (20%), phenytoin (31%), carbamazepine (33%), carbamazepine-10,11-epoxide (40%), trimethadione (31%), gabapentin (14%), pregabalin (11%), flunarizine (7%), KBr (18%), cannabidiol (14%), fenfluramine (4%), bumetanide (4%), clonazepam (22%), nitrazepam (10%), diazepam (7%), lorazepam (6%), midazolam (3%), amobarbital (21%), phenobarbital (16%), *^,#^ flumazenil (7%), allopregnanolone (7%), pregnanolone (6%), epipregnanolone (6%), 3-hydroxypregnan-20-one (6%), vitamin B6 (6%)
**Drug families and scaffold**	Imidazolidine (18%), ^#^ succinimide (10%), acetamide (7%), ^#^ 2-pyrrolidinone (7%), pyrrolidine (6%), tetrahydropyridine (6%), isoxazole (4%)
**Diagnostic marker**	Exametazime (10%), quinolinic acid (3%)
**GABA-Transaminase Deficiency Biomarker**	^#^ Succinimide (10%), ^#^ 2-pyrrolidinone (7%)
**Investigational**	Cyano-7-nitroquinoxaline-2,3-dione (5%)
**Inducers**	Flurothyl (37%), pilocarpine (25%), (+)-bicuculline (8%), pentetrazol (32%), MPTP (6%), bemegride (20%)

* Use with caution; ^#^ more than one role, obsolete.

## Data Availability

Data sharing is not applicable to this article as no new data were created or analyzed in this study.

## Abbreviations

The following abbreviations are used in this manuscript:

AED	Antiepileptic Drug
AMPA	Alpha-Amino-3-Hydroxy-5-Methyl-4-Isoxazolepropionic Acid
CNS	Central Nervous System
CNQX	6-Cyano-7-Nitroquinoxaline-2,3-Dione
CSF	Cerebrospinal Fluid
FDA	Food and Drug Administration
GABA	Gamma-Aminobutyric Acid
HMDB	Human Metabolome Database
ILAE	International League Against Epilepsy
KBr	Potassium Bromide
MPTP	1-Methyl-4-Phenyl-1,2,5,6-Tetrahydropyridine
NMDA	N-Methyl-D-Aspartic Acid
PET	Positron Emission Tomography
PLP	Pyridoxal Phosphate
S/N	Signal-to-Noise Ratio
SPECT	Single-Photon Emission Computed Tomography
VNS	Vagus Nerve Stimulation
